# Histological Study of Glandular Variability in the Skin of the Natterjack Toad—*Epidalea calamita* (Laurenti, 1768)—Used in Spanish Historical Ethnoveterinary Medicine and Ethnomedicine

**DOI:** 10.3390/vetsci9080423

**Published:** 2022-08-11

**Authors:** José Ramón Vallejo, José A. González, María Eugenia Gómez-Navarro, José María López-Cepero

**Affiliations:** 1 Departamento de Anatomía Patológica, Biología Celular, Histología, Historia de la Ciencia, Medicina Legal y Forense y Toxicología, Facultad de Medicina, Universidad de Cádiz, E-11003 Cádiz, Spain; 2 Grupo de Investigación de Recursos Etnobiológicos del Duero-Douro (GRIRED), Facultad de Biología, Universidad de Salamanca, E-37071 Salamanca, Spain

**Keywords:** *Epidalea calamita*, amphibian drug discovery, skin, histology, zootherapy, ethnoveterinary medicine, epistemological approach

## Abstract

**Simple Summary:**

Common toads, including the natterjack toad (*Epidalea calamita*), have been used since ancient times for remedies, and thus constitute excellent biological material for pharmacological and natural product research. After a previous analysis of the historical-folk therapeutic use of amphibians in Spain, a histological study was carried out to provide a complementary ethnopharmacological view through the analysis of the integumentary heterogeneity of the serous (venom) and mucous glands from two adult specimens. Plastic-embedded semi-thin sections showed that serous/venom glands are cytologically homogeneous in spite of their genetic and biochemical complexity, leading to a cocktail that remains stored until extrusion. On the contrary, mucous glands, working continuously, show a more complex cytological variation and regional heterogeneity, which suggests an adaptive variability, leading to an invisible topographic map of skin toxicity. Natterjack toad-based folk remedies are usually extracted from the whole animal as a therapeutic unit in ethnoveterinary practice. However, a new ethnopharmacological vision could emerge from the study of tegumentary regional variation.

**Abstract:**

Common toads have been used since ancient times for remedies and thus constitute excellent biological material for pharmacological and natural product research. According to the results of a previous analysis of the therapeutic use of amphibians in Spain, we decided to carry out a histological study that provides a complementary view of their ethnopharmacology, through the natterjack toad (*Epidalea calamita*). This species possesses a characteristic integument, where the parotoid glands stand out, and it has been used in different ethnoveterinary and ethnomedical practices. This histological study of their glandular variability allow us to understand the stages through which the animal synthesises and stores a heterogeneous glandular content according to the areas of the body and the functional moment of the glands. To study tegumentary cytology, a high-resolution, plastic embedding, semi-thin (1 micron) section method was applied. Up to 20 skin patches sampled from the dorsal and ventral sides were processed from the two adult specimens collected, which were roadkill. Serous/venom glands display a genetic and biochemical complexity, leading to a cocktail that remains stored (and perhaps changes over time) until extrusion, but mucous glands, working continuously to produce a surface protection layer, also produce a set of active protein (and other) substances that dissolve into mucous material, making a biologically active covering. This study provides a better understanding of the use of traditional remedies in ethnoveterinary medicine.

## 1. Introduction

Therapeutics throughout history has enabled us to compare the close relationship between the treatment of animal and human diseases over time [1,2,3,4]. We therefore find, for example, eminent pharmacologists and physicians in the 16th century who made excellent contributions by compiling knowledge from antiquity in both fields. Among the gallery of scientists, the following ones stand out: Dionisio Daza (1510–1596), Andrés Laguna (c. 1510–1511–1559), and Alonso Suárez (*Fl*. p. m. s. XVI) [5,6]. In the modern eras, the approaches of Rudolf Virchow (1821–1902) and William Osler (1849–1919) are remarkable for developing the concept that human health and animal health are inextricably linked. This idea is currently on the rise due to the spread of emerging diseases as a consequence of interaction between human beings, animals, and ecosystems [7,8,9,10,11].

On the other hand, the traditional systems for caring for, healing, and maintaining the health of animals and humans, known as ethnoveterinary medicine (EVM) and ethnomedicine, respectively, are embedded in all civilisations and cultures. Hence, there now exists an investigative mentality that seeks to relate traditional or popular therapeutic remedies that are part of the arsenal of human and veterinary ethnomedicines, to highlight them [12,13,14]. Moreover, the integration and eclecticism of this traditional knowledge (TK), preserved over time but also evolving, can be a source of information for translational and biomedical research [15]. In this way, as demonstrated in ethnopharmacology, natural resources are a shortcut to the discovery of new medicines because of their biologically active components [16,17,18]. Although to a lesser extent than plants, a growing number of investigations these days is aimed at the creation of new drugs or chemically active products from animals, or structures and tissues derived from these organisms [19,20,21,22]. On the other hand, in many developing countries, people, especially in rural communities, still rely on traditional medicine and EVM for basic health care, and animal-based folk remedies persist. Hence, TK in different cultures and the analysis of historical texts, providing cultural studies on the medicinal use of animals, are useful as a basis for pharmacological research, to validate these uses and allow the development of new medicines [23].

However, one of the most interesting aspects is the use of animal venoms as candidate drugs, new drugs, and therapies [24,25]. In this respect, amphibians are of particular interest (this animal group has been used since ancient times for remedies that have survived until the present day), and more particularly the anurans of the family Bufonidae, which constitutes excellent biological material for pharmacological and natural product research [26]. Bufonids have been traditionally used in the EVM of Brazil, China, and Korea [27,28,29]. In relation to European ethnomedicine, in Spain there is an extensive review based on Pliny the Elder, Pedanius Dioscorides, and other popular therapeutics [30]. Throughout history, tribes and traditional human communities have used the skin and the secretions of their parotoid venom glands to treat a wide range of diseases and troubles, such as haemorrhages, inflammation, poisonous animal bites and stings, skin and stomach disorders, as well as infections or various types of cancer [31,32,33,34]. Thus, as representative data from a historical point of view, in 1813 Bell [35] described that when faced with a scorpion sting, it is advisable to kill the animal and apply it to the injured part, or cover it with a toad or with another poisonous dead animals.

According to the analysis of the therapeutic use of anurans in Spain [30], it follows that the species *Bufo bufo*, *Epidalea calamita*, and *Pelophylax perezi*, which are found throughout Spain, are the most culturally important in this country at present. For this reason, we decided to carry out a histological study that provides a complementary view of the ethnopharmacology of amphibians, through the species *Epidalea calamita* (previously named *Bufo calamita*). It is a unique species due to the taxonomic family to which it belongs (Bufonidae), with a characteristic integument where the parotoid glands stand out; it also stands out for its ecological, ethological, and biogeographical characteristics. In fact, *E. calamita* is a Palaearctic species native to south-western and central Europe with a wide distribution range in Europe [36].

The natterjack toad is an amphibian of medium size, with a wide range of sizes; the adults measure about 5–6 cm in length, with a maximum among females of around 9.5 cm. It can be distinguished from the common toad by a conspicuous yellowish or greenish dorsal midline that usually runs longitudinally along the body from the base of the eyes to the cloaca. It is robust in appearance, with a squat and very warty body. The iris is metallic yellow and the parotoid glands are rounded and parallel. They have relatively long legs, which give them their distinctive gait, in contrast to the hopping movements of many other toad species [37]. It is able to survive in relatively dry areas provided it has at least one pond in which to breed. It is a good coloniser that quickly occupies pools in abandoned gravel pits or quarries, drinking fountains, or troughs, etc. Its short larval period allows it to exploit temporary shallow pools of very different sizes [37]. They show a clear preference for breeding in pH-neutral aquatic environments, where their larvae have optimal development; however, they are able to tolerate only certain levels of acidity in the water [38]. It is one of the most common anurans in many of its distribution areas in regions such as the Spanish mainland [39,40,41,42]; however, in other regions of Europe, it is seriously threatened; for example, it is regionally Red Listed as Endangered in Ireland [43]. Worldwide, it is in the “Least Concern” (LC) category of the *IUCN Red List of Threatened Species* (v. 2015.3), showing a decreasing population trend. In Spain the species is listed in the LC category. Among the threats to this amphibian is the alteration to its habitats; for this reason, in countries such as Ireland, pond restoration and reintroduction projects are being carried out with ex situ release programmes [44]. As to the pressure motivated by captures for the practice of local healers or popular medicine, this can be considered negligible, especially in Spain where popular zootherapy has been relegated and in practice these are relics or “survivals”, or vestiges of the past. Nevertheless, TK of them is useful for both prospecting candidate drugs and for establishing relations with regions such as Latin America, where the Spanish passed on animal remedies, and the philosophical bases of the humoral system, which is still in force.

### 1.1. Medicinal and Veterinary Practices Based on Epidalea Calamita

Folklore, history, and anthropology, both medical and veterinary, as well as social and cultural, can be very useful in determining the species that make up a popular therapeutic arsenal. As already mentioned, in Spain there is currently an important review of the therapeutic use of amphibians, where medicinal species have been identified [30,45]. So, in accordance with the methods and objectives of the Inventario Español de los Conocimientos Tradicionales (IECT, the Spanish Inventory of Traditional Knowledge), the zoological species and groups that have been used as therapeutic resources in this country are being documented and identified [46,47,48]. To this effect, there is currently a list of therapeutic remedies based on the use of nine species of amphibians—two caudates (urodeles) and seven anurans—compiled through a narrative review [30,45]. Table 1 shows the ethnoveterinary and ethnomedical practices based on the use of *Epidalea calamita*, the subject of this work, and their medicinal uses, which can also be shared with the common toad (*Bufo bufo*) [30,45]. In this regard, Ceríaco [49] notes that, in Portugal, the use of toad slime against herpes is a remedy extracted from three different anuran species: *Bufo bufo*, *Bufo calamita*, and *Pelobates cultripes*. This viral condition, which will be discussed below, has also been treated in Spain with *B. bufo* and *E. calamita*.

In the treatment of infectious diseases in Spanish EVM, the studied species has been widely used. Ruminant hoof disease, known as scald or hoof rot, caused by the anaerobic bacterium *Fusobacterium necrophorum*, used to be treated by putting a natterjack toad sliced in half on the affected leg [50]. Treatments for the skin and subcutaneous tissue are also noted. Rubbing the affected area with the skin of this toad was a useful remedy for skin ulcerations in animals in the province of Badajoz [52]. On the other hand, in Fuenteguinaldo (Salamanca), when people came across a dead dry natterjack toad, they took it, crushed it, and covered “cancerous wounds” (that would not heal) of domestic animals with the powder obtained [51]. If we look at the treatment of human diseases, we can see that amphibians have been widely used for zoonotic bacterial diseases, such as the case of anthrax. For example, it was common practice to place a live toad on grain stalks, e.g., wheat and barley, hold it in place with a bandage, and in this way it was kept there until it died, which happened after eight days. By then, according to informants in the Basque Country, the anthrax would be cured [53]. For the treatment of brucellosis, the technique used in Valencia was to leave a toad loose in the patient’s room for two days; after this time, it was killed and put in a poultice on the patient’s chest [50].

Regarding the respiratory system (upper respiratory tract disorders), the treatment of tonsillitis is notable. Specifically, in Lalín (Pontevedra), a decoction of the herbaceous plant known as “oreja de liebre” (possibly “lampwick plant”, *Phlomis lychnitis* L., Lamiaceae) is used [54]. Nevertheless, in other Galician areas, in order to accelerate suppuration, the effect is doubled by including an opened natterjack toad in the potion [55]. In tonsillitis, an opened toad also is applied to split the tonsils [54]. For headache, there is a remedy by placing on top of the patient’s head a recently caught toad, which took care of eliminating the pain [56] (p. 595). Other significant data focus on the skin and subcutaneous tissue. For example, in cases of warts in Galicia, the belly of a live toad was rubbed on the skin [50]. In general, warts were rubbed in an “unspecified” way on the skin [57]. Herpes zoster was also cured through a ritual that is interesting from an anthropological point of view, although it is not an empirical remedy. The practice, as performed in Albox (Almería), consisted of taking a toad and passing it alive nine times over the affected part (both upwards and downwards). It was then hung up in a tree to dry until it died. The same action was repeated with another two toads (making a total of three). The informant says that the first toad came out of the operation swollen and “burning like fire”. The second toad was somewhat less swollen and hot, and the third was less so [58]. Venomous animal bites (spiders, snakes) were also treated by rubbing them with toad skin [57].

### 1.2. Bioactive Constituents and Candidate Drugs

Currently, the scientific literature has numerous studies and extensive reviews that document the pharmacological value of amphibians and their benefits in biomedicine [34,59]. Due to their interest in the history of pharmacology and therapeutics, we can highlight, among others, the studies by Kiss and Michl (1962) on the species *Bombina variegata*; thanks to them, the antibiotic properties of the bombinin peptide were determined [60]. In the 1960s, too, Daly’s work stands out for his contribution to the identification of the compound ABT 594 (Abbott Laboratories) from *Epipedobates tricolor* as a non-opioid analgesic between 30 and 100 times more powerful than morphine [61,62,63]. It can be stated that the compounds synthesised by amphibians are quite numerous and diverse in chemical composition, but most of them include biogenic amines (adrenaline, noradrenaline, bradykinin, or histamine), steroids (bufadienolides and bufotoxins), alkaloids (batrachotoxin or tetrodotoxin, mostly in the Dendrobatidae family), peptides, and proteins [59]. In recent years, pharmacological and chemical studies on amphibians are still relevant, and can be considered a hot topic due to the interest in traditional Asian medicines or from regions such as Central and South America [26,34,64]. However, the contributions on the medicinal use of amphibians in specific European popular medicine and/or EVM are occasional. Ethnopharmacological studies in Europe are primarily focused towards the use and properties of medicinal plants, in such a way that works on the therapeutic usage of animals are scattered [65,66] or limited to a few articles on animal-based remedies in ethnomedicine and/or EVM [45,46,47,48,67]. Hence, for example, we currently do not have specific projects that register the bioactive components related to the medicinal use of species such as the ones we are dealing with. However, its use in European folk medicine and EVM encourages studies to learn about and isolate the chemical structures present in *Epidalea calamita*.

In any case, considering the extensive existing documentation, it can be affirmed that amphibians present a promising veterinary and pharmacological potential as a source of drugs. On the other hand, it should be noted that there are at least 47 species of amphibians used in traditional medicine around the world. Among them, the family Bufonidae stands out with 15 species, followed by Hylidae and Ranidae, with 8 taxa, and Salamandridae, with two [68]. In the sphere of EVM, the species recorded in the literature are all restricted to Bufonidae [68]. As a result, there is a tendency to validate traditional uses, looking for chemical profiles, pharmacological activity, toxicity, and quality control in Bufonidae, where bufadienolides, such as bufalin, cinobufagin, resibufogenin, and telocinobufagin, are found, which are the major active compounds derived from toad skin [64,69,70]. Although the traditional use of toad skin to cure wounds and skin ulcerations in livestock involved the death of the specimens, it is appropriate to note that the skin and glandular secretions of toads contain several classes of chemical compounds, such as the bufadienolides mentioned earlier, in which antitumour and anti-inflammatory activities have been found [69]. In general, the toxins and bioactive chemical constituents of the skin and secretions of the parotoid glands of the bufonid species can be grouped into five categories: (i) the guanidine alkaloids; (ii) the lipophilic alkaloids; (iii) the indole alkaloids; (iv) bufadienolides; and (v) peptides and proteins isolated from the skin and gastrointestinal extracts of common toads. For this reason, certain popular treatments can be justified after validation, as their bioactive secretions may have antimicrobial properties, protease inhibitory and anticancer properties, as well as being active at the neuromuscular level [34]. It should be noted that perhaps the best documented cases are Asian native toad species (*Bufo gargarizans*, *B. melanostictus*) from which the “toad venom” (or *Venenum bufonis*) used in traditional medicine to treat local inflammations, sinusitis, toothache, and cancer sores is obtained [71,72,73,74]. This traditional remedy, also known as *Ch’an Su*, is being used in health systems where traditional medicine coexists with biomedicine in clinical therapy for heart stimulation, treating tuberculous fistula, diuresis, neurodermatitis, toothache, as an antitumour compound, and as a painkiller [59]. This is the consequence of an intense investigation of traditional Asian remedies where problems of cytotoxicity, bioavailability, or therapeutic dose are also contemplated [74,75]. Among the components of remedies based on species of the genus *Bufo*, there are well-known active ingredients, such as gammabufotalin, bufalin, cinobufat alin, cinobufagin, resibufagenin, desacetylcino bufotalin, cinobufagin-3-hemisuberate, 1-hydroxybufalin, 12-hydroxyl-resibufogenin, 19-oxobufalin, desacetylbufotalin, arenobufagin, telocino-bufagin, desacetylcinobufagin, cinobufaginol, and desacetylcinobufaginol, as well as other new bufadienolide and novel bufalin derivatives [26,64,74,75].

Other well-studied bufonids are *Bufo marinus* (now *Rhinella marina*) and *B. bufo*, from which bufotalin and bufotoxin have recently been isolated [26,34,59,64]. Among these species, it is of special interest for our work to dwell on the medical importance of the common toad (*B. bufo*), as there are data on its popular use both in Spain [30] and Portugal, where, for methodological reasons, on some occasions they have been considered as the same remedy as *E. calamita* [49] (p. 337). Thus, its skin secretion has very strong antimicrobial activity against *Staphylococcus epidermidis*, *Enterococcus faecalis*, and *E. faecium*, and activity that can be characterised as moderate against *S. aureus* [59]. Regarding its anticancer properties, cytotoxic activity has been seen in human alveolar adenocarcinoma, human colon colorectal adenocarcinoma, HeLa human cervix adenocarcinoma, human mammary gland adenocarcinoma, human pancreas adenocarcinoma, human prostate adenocarcinoma, and human glioblastoma astrocytoma [59]. However, as already mentioned, there are currently no data on the active components from the species under study. Therefore, it is necessary and very interesting to promote scientific studies on the possible uses of these compounds in the natterjack toad for the maintenance of animal health [45,64].

### 1.3. A New Ethnopharmacological View through the Integument

**Figure 1:**
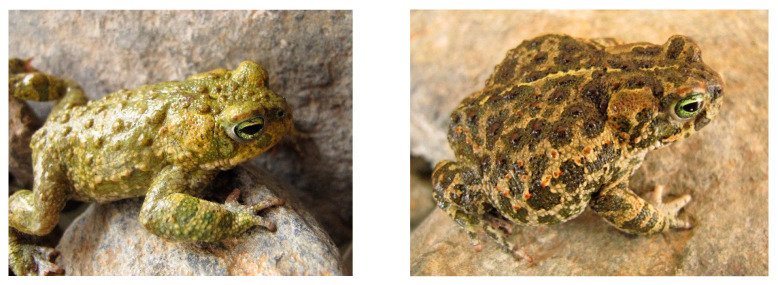
The images show the great variability in colour of the back of the natterjack toad that can also have a clear and light, narrow mid-dorsal line (photos by J.R. Vallejo).

The skin of *Epidalea calamita* is rough and with many pores, with protruding warts, sometimes stained red, and also porous. On the back, greenish tones predominate, sometimes mixed with brownish, reddish, or yellowish colours. It is important to point out that individuals from the same population vary greatly in their colouring [76,77]. Thus, the structure and topography of the skin of this toad species in terms of colouring, distribution of glands, etc., shows a zonal variability that can be explained by ontogeny or developmental biology and where its parotoid glands stand out (Figure 1). However, from the EVM and ethnomedicine point of view, the empirical data obtained lead us to consider this species as a zootherapeutic unit, since in almost all cases the whole animal is used [30,45]. On the other hand, the zonal variability and therefore their glands do not act synchronously (that is impossible) but have cycles of activity and rest and are therefore not strictly equivalent in terms of composition and pharmacological activity. So, when using the whole animal as a source of bioactive substances, the analysis of this variability could be considered irrelevant. However, the histological study of their glandular variability can allow us to understand the stages through which the animal synthesises and stores its heterogeneous glandular content according to the areas of the body and according to the functional moment of the glands. For this reason, the main objective of our study is to study the variability that they present for two reasons: (1) the histogenetic mechanisms that condition the formation of glands of one type or another, and probably regulate their size; and (2) the variability produced by the stages of glandular functioning since they do not all function or store substances synchronously, nor do they all have to occur at the same functional moment. Moreover, by using histomorphological techniques, we aim to provide a new ethnobiological perspective, using an interdisciplinary approach that can contribute to a better understanding of the therapeutic use of amphibians by human communities throughout history. In this way, we wish to contribute a new epistemological approach to ethnopharmacological research, since by studying this animal, and therefore other toads, it will be necessary to take into account not only the “typical” composition of fully developed glands but also those at another stage of their functional cycle. This approach may be useful considering that qualitative and quantitative tools based on MS spectrometry and nuclear magnetic resonance spectroscopy now exist to study the complex secretions of bufonids [34,78].

Finally, we hope that our contribution will contribute to a better understanding of the skin of this species and will be of use to scientists involved in the conservation of wild populations, captive breeding, and reintroduction in decimated populations [43]. Undoubtedly, the alarming situation of amphibian populations requires an interdisciplinary effort in which data derived from research with different approaches, aims, and objectives can be transferred to the conservation of biodiversity. Next, after the methodological section, the histological study is described according to the epistemological approach indicated.

## 2. Materials and Methods

Two adult specimens of the studied species were collected on 9 February 2021 in the municipality of Badajoz during a nocturnal transect on a local road on a night following rain. The weather conditions were suitable for typical amphibian migratory flow. Transects are part of the amphibian sampling and census method that was carried out on foot or by car.

It should be noted that there is a high mortality of amphibians on the road due to their movements during the breeding season [79,80]. Taking advantage of this circumstance, both animals collected were roadkill. From a methodological point of view, it should be noted that tissue integrity is usually suitable for microscopic study for many hours (up to 24 h) after death. In fact, this is the rule that allows us to rely on the histopathological diagnosis that arises from clinical autopsies. Thus, the parts of crushed tissue (one leg, for example) can be easily identified and discarded and are not used for histological study.

The specimens were fixed in 10% formol (4% formaldehyde solution) from the moment they were found. The abdomen midline was opened longitudinally to allow the fixer to reach the internal organs. The post-mortem interval before fixation was unknown but overall tissue quality was qualitatively assessed by skeletal muscle (minimal) changes in fibrillary architecture and good cytological detail in most organs. Crushed corpse parts were not used for microscopy. Only males were available and gonadal sex was confirmed in the paraffin blocks of testes made from internal organs (not shown) for a routine histopathological necropsy study. To study the tegumentary cytology, a high-resolution, plastic embedding, semi-thin (1 micron) section method was applied. Symmetrical (left-right) skin regions were sampled from the dorsal and ventral sides in a cranio-caudal order. Up to 20 skin patches were processed for each animal. Parotoid glands were embedded separately.

Tissue samples from the tegument were dehydrated progressively in cold acetone (at 4 °C), infiltrated in glycolmethachrylate (GMA) solution (20 mL hydroxyethylmethacrylate monomer + 2 mL ethyleneglycol monobutyl ether) and catalysed with benzoylperoxide (720 mg/100 mL). Continuous rotation for two days allowed a full infiltration; then the tissue fragments were oriented and positioned (glued) over a previously polymerised methachrylate base in a tightly closed polypropylene flask and covered with new catalysed GMA (freshly prepared). Polymerisation was performed under fluorescent UV (black light) for 2–3 days. Blocks were trimmed by polishing the lateral and frontal sides, oriented to make transversal skin sections, and glued to the stubs, to put them in the microtome holder. Semi-thin sections (1 and 2 µm thickness) were cut with a tungsten carbide knife, at low speed, in a Leica RM2155 motorised microtome, floated over an ammoniacal cold water bath, mounted on clean slides and dried over a hot plate (60 °C) for complete adherence. Adjacent sections over the same slide were true serial sections containing slices of the same cells. Most sections were stained with 1% toluidine blue (C.I.52040) dissolved in 5% borax), usually or less than for 1 min., and washed and dried again over the hot plate. Other staining methods were applied to the adjacent slides: (1) Heidenhain’s iron haematoxylin; and (2) periodic-acid Schiff (PAS) staining. Cytological details were imaged through 63× (NA1.25) and 100× (NA1.30) oil-immersion objectives using a Leica DM2500 microscope adjusted for Köhler illumination to maximise the resolution. Microphotographs were taken with a Leica DFC495 CCD camera and were saved as .tiff files at a 3264 × 2448 pixels and 2176 × 1632 pixels resolution. Some digital microphotographs were adjusted when necessary for brightness and contrast (only histogram correction) with ImageJ (v1.53Q, downloaded as FIJI distribution, release 2.5.0). Several high-magnification fields were joined with the stitching plugin (ImageJ) to make composite images of large areas without losing resolution. A calibrated (10 µm-interval) micrometre objective was used to set the scale in the software and perform accurate measurements. Image analysis of the granule size distribution was performed to provide the only available morphological correlation for the content variability of (serous) venom glands.

## 3. Results and Discussion

**Figure 2:**
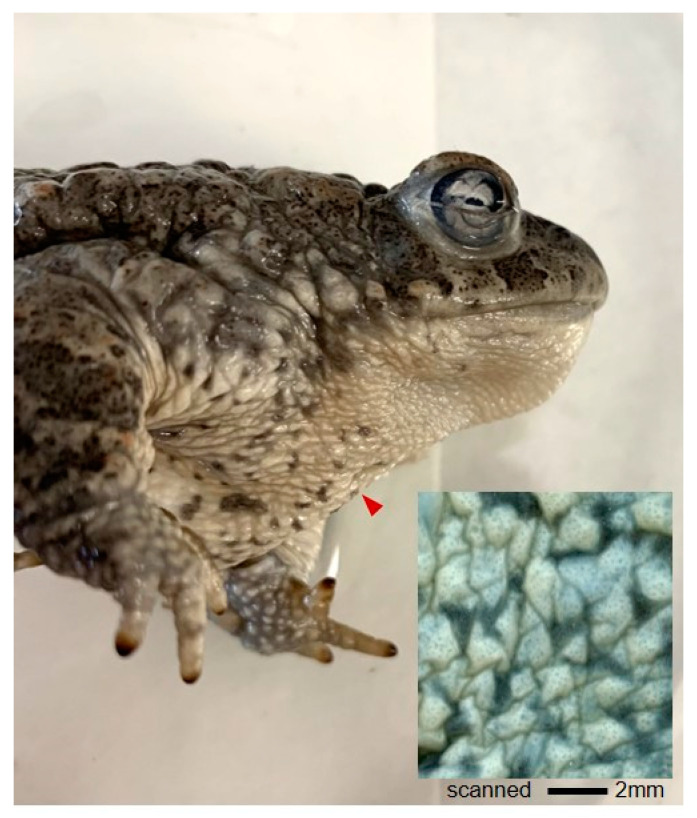
Characteristic aspect of dorsal and ventral tegumentary surfaces and pigmentary pattern. Ventral skin (inset) with smaller cystic serous glands, less relief, and lighter pigment density than on the back, allowing a better binocular observation of the light-reflecting pattern and individual melanophores on a surface view (photos by J.M. López-Cepero).

The individuals studied show the typical skin of the taxon. *Epidalea calamita* skin shows wart-like, dome-shaped elevations in the dorsal surface that correspond to dermal glands but still allow visualisation of the pigmentary subepidermal pattern by binocular microscope or epi-illumination. A digital scanning of the ventral surface (Figure 2) shows the reflected light pattern obtained from three chromatophore subepidermal layers (xantophores, iridophores, and melanophores). The cobblestone appearance is misleading because the skin surface makes irregular folds and grooves, although the epithelium is flat, continuous, and mucus-wet under microscopy. A binocular microscope can see individual melanocytes and pigmentary spots. Histological processing for semi-thin plastic sections provide a good resolution and enough cytological detail to interpret tissue processes underlying regional variability.

### 3.1. Tegumentary Architecture

**Figure 3:**
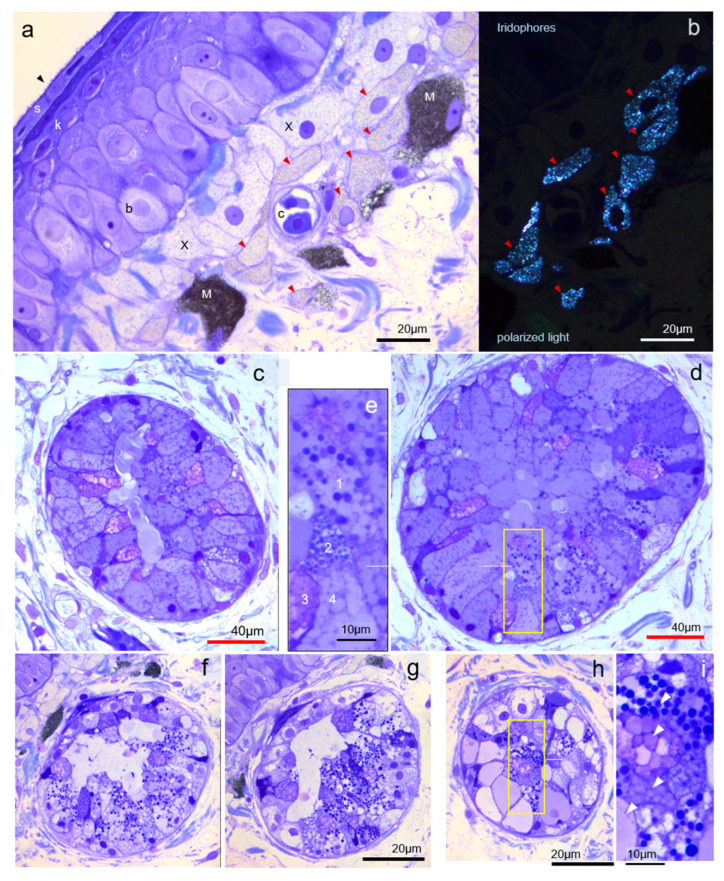
Semi-thin sections of tegument epithelium and mucous glands. Toluidine-blue stained. Surface epithelium and pigmentary layers architecture (**a**); iridophores (**b**) of the previous section (arrowheads) under polarised light. Common (**c**) and large (**d**) mucous glands and morphology of the secretory granules (**e**). Near-adjacent sections (**f**,**g**) of the smaller glands show patchy clonal (homogeneous type) cell areas in the adenomere but they still contain every type of secretory granule (cell types) (**g**,**h**,**i**) (for legend references, see the text) (photos by J.M. López-Cepero).

The tegument architecture of *Epidalea calamita* showed the already extensively studied epidermis (Figure 3a), including a basal layer (b), was made of large cells giving origin to more superficial keratinised (k) layers, and a last dense compacted thin layer just under the epidermal surface (s), which is made by flattened cells with a microvilli border (black arrowhead) covered with the protective mucous secretion. Just under a thin basal lamina, a single or double row of xantophores (X), intermingled with a loose mesh of collagen fibres, is the first light-filtering pigmentary layer. Cytoplasm is foamy and devoid of pigment after acetone dehydration steps in the embedding procedure. The second chromatophore layer is a single row of flattened iridophores (red arrowheads) filled with reflective platelets of purine (guanine) crystals visualised under crossed polarisation filters (see Figure 3b). At the same level, distributed at irregular intervals, melanophores (M) containing a dense mat of extremely small (0.2 µm) pigment granules (melanosomes) send occasional branches upwards, sometimes entering the epithelium (not always) and delivering pigment to keratinising cells (only in some areas). These combined layers produce a surface colour by selective absorption and reflection and can show functional (structural) adaptation to changes in the colour spectra over short time intervals in many amphibians. The most superficial skin capillary network (c) is always under a xantophore layer and positioned at the same level as iridophores and melanophores.

### 3.2. Mucous Gland Architecture and Cytology

Deep in the chromatophore layers, a connective tissue with loose collagen fibres and most of the skin vascular network contains mucous glands all over the skin surface with evident variability in size, morphology, and functional stage. A prototypical, common gland (Figure 3c) is ovoid or spherical, about 100–120 µ Ø, with a small alveolar lumen filled with amorphous secretion. Larger glands can reach ×2 Ø (up to tenfold volume) (Figure 3d) and change the proportions of the cell types. In common glands, secretory cells constitute a single crowded cell layer with central or basal nuclei and the large apical cytoplasm filled (under tension) with granules of different sizes and content that define the four cell types. When stained with toluidine blue (and using only morphological criteria), fully differentiated, adult “mucous” cells are always from two types/lineages. The first one has abundant, rounded, or slightly elongated cells with large granules (unknown content), a dense cytoplasm, cell bodies compacted by pressure against the neighbours and pushing and extruding the apical part of a second type, are less frequent, caliciform-type cells, metachromatically stained with TolBlue (magenta colour), and still anchored to the basal membrane. Nevertheless, all mucous glands also contain abundant serous (granular) cells not seen in routine histopathology with HE-stained paraffin sections. Secretory granules stain strongly with TolBlue (and iron-haematoxylin). Accumulated secretion in the lumen is always devoid of granules, suggesting that they are dissolved (or processed) quickly when all components are mixed. Four types of terminal-differentiated cells (Figure 3e) can be identified by their secretory content.

Mucous glands tend to accumulate cells filled with secretion and store a large amount of loaded cells (and only some secretion in the lumen). When glands grow in size, they compress the adjacent connective collagen network. They are larger and more frequent on the dorsal (back) side. The smallest glands (most glands in the ventral side) exhibit a different structure and cell population kinetics: they have the same cell types but more abundant serous (granular) cells, and their spatial distribution suggests a clonal structure of secretory epithelium (patches of cell types) (compare Figure 3f,3g, same gland). They also show a variable (stochastic?) proportion of serous/mucous cells in every gland (Figure 3h), although maintaining all the cell types already described (Figure 3i). These facts suggest a single stem cell for mucous glands (either large or small). The proportion of cell types could be adjusted (as usual) by modulation of the amplification compartment (by growth factors or local signalling) or by resident time in the gland before extrusion. The smallest glands show a larger variability in cell composition and probably follow a secretory cycle (or aleatory cycle) where the mucous and serous secretions are not synchronised. Serous (granular) cells of mucous glands are not obviously analogous to granular secretion from venom glands but they must be closely related to the chemical protection of the mucous layer (antibiotic, antifungal, etc.), giving an opportunity to provide a heterogeneous surface map of mucus properties.

### 3.3. Variability and Functional Secretory Cycles

**Figure 4:**
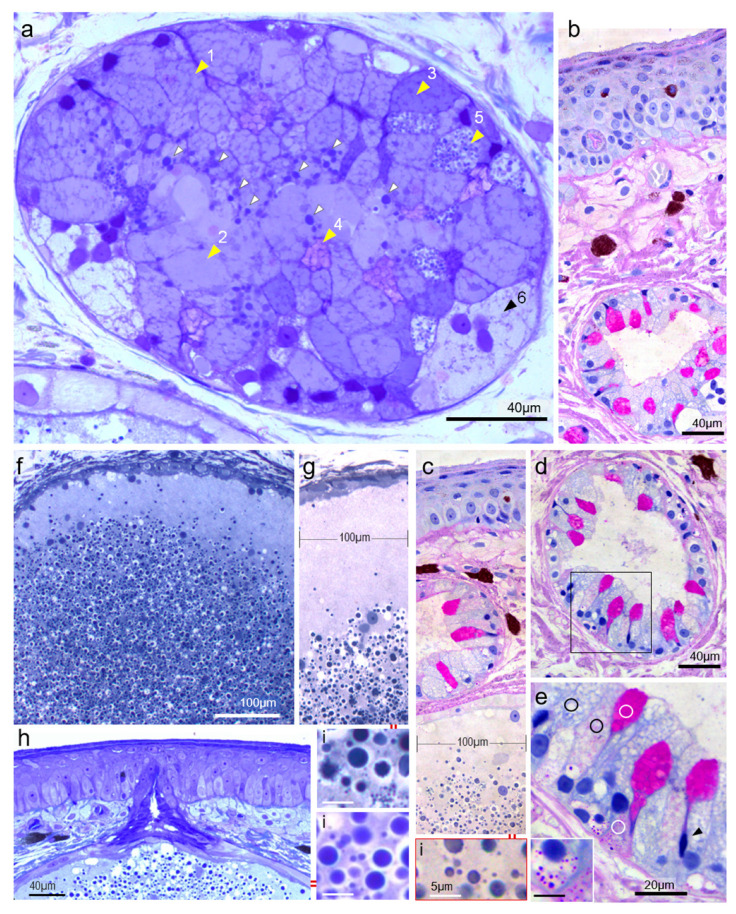
Large adenomeres contain all cell types and differentiation stages (see text) (1 µm section, TolBlue) (**a**). Only a subset of mucous cells (**b**) are glucoprotein PAS+ secreting cells, whereas adjacent ones are PAS- (**c**) (2 µm sections). All PAS+ cells are caliciform-type cells (**d**) interspersed between other cell types (**e**), storing secretion in their apical pole, and immature basal cells with PAS+ secretory granules (inset). Low power (**f**) serous (venom glands), (2 µm section, iron haematoxylin) with a peripheral syncytium layer devoid of granules (**g**). Keratinised lumens of the secretory ducts (**h**) maintain a closed compartment under pressure. Granule staining showing similar aspects (**i**) and size distributions with iron haematoxylin, PAS, and toluidine blue methods, revealing their predominant protein nature (in sections) (photos by J.M. López-Cepero).

Cytological variability due to immature stages of adult (terminally differentiated) cells can only be settled by immunohistochemical markers of the cell lineage that were expressed well before overt differentiation. Nevertheless, some details could be interpreted as stages of cell biography. When a large population is reached inside a gland because the elimination rate is lower than the proliferative and differentiation kinetics, it is more probable to find many intermediate stages. The section shown in Figure 4a is one such example. The most abundant mucous cell is filled with compacted material and has a rounded basal nucleus ranging from a foamy aspect (1) with faceted polygonal granules to a homogenous (amorphous) secretion that accumulates (extracellularly) as a large mass occupying the lumen (2). A few similar cells in the basal position showed a slightly more basophilic cytoplasm (3). Their secretion constitutes more than 80% of the surface area and (by stereological rules) must be 80% of the volume of the enlarged (quiescent, non-secreting) alveoli, but only one third is extracellular material. Whole cells (perhaps only apical cytoplasm) seem to fall apart as a secretory mass in a reminiscent way of apocrine secretion. Mucous metachromatic cells (4) are few and isolated and they do not seem to lose integrity to extrude the mucus in a classical merocrine pathway. Its material cannot be stained extracellularly, in contrast with the large serous granules (protein content) that remain visible only in the peripheral part (white arrowheads) of the centrally stored mucus, probably dissolving in contact with it. Granular serous cells filled with small identical granules (5) that do not fuse for enlarging, are the less abundant volumetric components of the secretion. Chromophobe and (sometimes) binucleated cells (6) can be seen only in heavily loaded glands, suggesting a non-functional regressive change. From this picture, the population dynamics in the closed compartment of a saving gland suggest a higher production rate of homogenous masses of mucus with two components, enriched with a lesser amount of protein secretion arising from two different differentiated cells. Periodic acid–Schiff (PAS) histochemistry indicates that only caliciform-type cells are stained (Figure 4b) and this mucus corresponds to the slightly metachromatic cells with TolBlue. Basal membranes and collagen are slightly PAS+. By contrast, the serous gland content is not PAS+ (Figure 4c). In common glands (Figure 4d), continuously secreting, an empty alveolar lumen shows that internal epithelium is distributed as patchy areas of different stages in the secretory cycle (selected area Figure 4d). Fully differentiated loaded mucous cells show different shades that (surely) represent progressive maturation of mucus up to the typical foamy aspect that precedes their extrusion. Adjacent caliciform, fully mature cells and some basal PAS+ small cells, along with the absence of graded intermediate phenotypes (Figure 4e), indicate that differentiation and cell turnover of this cell type is really fast, and the resident time is much shorter than the other abundant neighbours’ mucous cells, taking more time to synthesise an equivalent volume of stored secretion. Reasoning in this way, cell abundance does not mean more secretory daily production.

### 3.4. Venom Gland Architecture

Serous glands have a common aspect, with differences related only to size, growing time, and intra-alveolar processing or maturation of secretion (Figure 4f,g). These glands are cystic spaces enlarging for a long time until a discharge is provoked by mechanical or other stimuli not well characterised. Their growth pushes against adjacent glands (see Figure 4c), the collagen network, capillaries, and overlying skin, to form large bumps on the back of the animal. The excretory duct crossing all epithelial layers, made of keratinising cells, make a plug, sealing the exit pore (Figure 4h). Along their basal membrane, they also have a single layer of flattened myoepithelial cells that could be responsible for an (moderate) increase in pressure, just enough to open the pore and allow the material stored under pressure to escape [81]. The epithelium acquires a syncytial character and all granules remain isolated, sometimes fragmenting themselves, but they are localised only in the central part, leaving a clean peripheral mass of syncytial cytoplasm where nothing remains for long, with not even displaced nuclei moving themselves into the granular mat. Measuring granule size is not reliable because of their shrinking with all kinds of fixation that leaves a halo effect. Measuring and counting them by image analysis shows that they follow a potential law (not shown). Nearly all the basic colourants (including iron-haematoxylin) provide a strictly similar staining (Figure 4i), and relative optical-density distribution upon size. Hence, morphological data are not an index of processing or maturation of secretory content, although biochemical and genetic data indicate that specific venom precursor proteins are produced just to be cleaved to active peptides [82]. The chemical modification or degradation of other venom components upon time cannot be discarded but published evidence is not available [83].

### 3.5. Histogenetic and Evolutionary Basis of Variability

In all developmental systems, histogenetic (local) mechanisms are translating, buffering, and integrating the genetic variance (mutations or genetic background) into phenotypic variation at the tissue level [84], in a context where systemic factors (e.g., hormones) or environmental influences are used for (or against) the optimal pattern of tissue architecture and function. Variation in the sensitivity of developmental systems to genetic or environmental perturbations is named “canalisation”, and how predictable the developmental outcome is (in spite of these influences) is referred to as “developmental stability”. Both concepts set the frame to look for mechanisms and to interpret the effects of an available genetic background into the phenotypic result at the tissue (and organismal) level [85] (p. 529). The complex hierarchical relationship between genetic variation, developmental mechanisms, phenotype results, and tissue plasticity (functional adaptation) converge into the histogenetic process; but, later, at higher levels, the functional performance of organs and integrated physiology and behaviour contribute to the homeostasis, survival, or reproductive fitness of individuals. A high variability in skin adjustments of the basic developmental programme seems to be necessary to explain the evolutionary variation in the tegument properties between species, adapted to many different aquatic and terrestrial habitats, ecological pressures, and resistance to infectious, parasitic, or fungal diseases [86]. The structural and mechanical barrier of the tegument is reinforced and personalised by a mucous, chemically doped surface, provided by the glands.

Following the approach stated in Section 1.3, by providing a new ethnopharmacological view of the skin of this taxon, it should be noted that the tegument of amphibians is an essential and multifunctional organic surface [87]. In addition, there are abundant descriptions and comparative histology of the amphibian tegument; chromatophores and glands are also available, at both the optical and ultrastructural level [88,89]. Thus, the tegument of amphibians exhibits a topographically variable colour pattern by several types of pigment cells derived from the neural crest, with different composition, absorption, and dispersion spectra [90]. These patterns emerge during embryonic (larval) development, metamorphosis, and adult tissue adjustments by plasticity. Developmental mechanisms provide a great potential for phenotypic variation that can be filtered and fixed through natural selection. The skin also contains a series of glands destined, on the one hand, to lubrication and defence by means of antifungals and antibiotics of the moist surface of the epidermis [91,92], but also other glands specialised in the accumulation of toxic substances with a defensive function [93,94,95].

The distribution pattern of skin toxicity over the animal’s surface is not homogeneous. The chemical composition of stored venom has been analysed. There are no data on the differences in the toxicological composition of glands in different topographical regions of the tegument. The morphological aspects of venom glands offer little clues about their biochemical composition. Mucous glands, on the contrary, exhibit a wider phenotype modulation that follows a topographical pattern. Conventional histology in paraffin sections offers a low resolution with respect to cellular details and the composition of the various glands. The study of semi-thin sections of glycol methacrylate (GMA) allows us to describe with much greater detail and resolution the cytology of the cutaneous glands, and therefore the structural basis of the topographical map of secretion and toxicity. The most relevant aspect of gland variation is that it arises by modulation of a histogenetic developmental programme, and the careful interpretation of structure and cytology can shed some light on what processes have been necessary to get the current gland phenotypes and their range of local variation.

The basic structure of mucous glands (in tegument) is presumed to be homologous (sharing a common ancestor origin) for all amphibians, with little evolutionary change in histogenetic mechanisms but a wide range of interspecies variation by changing the cell differentiation styles and secretory synthesis. Its histogenetic and evolutionary relationship with serous (venom) glands is not clear, although great differences in secretion chemistry (from skin extracts) have been described [96] and related to a functional role (antiseptic, antibiotic, antifungal, toxic defence, etc.) or to environmental adaptation [97,98]. Most studies of venom gland chemicals and their biological functions, toxicity, or pharmacological activity were performed by using the whole extract from all over the skin surface or from selected areas (parotoid glands) with a high content of stored secretion [99]. Progressive changes in toxic compounds by processing or maturation of stored secretion have not been addressed [100]. Such venom cocktails, extracted as a whole, do not envisage the glandular topographical, biochemical, or functional variability, in both secretory composition and biological properties. Just like the pigmentary skin pattern exhibits a topographical variation, the glandular pattern distribution and content do not need to be homogenous, and they can be suspected to provide the skin surface with a protective and topographical toxicity map. Such functional variation must be correlated with subtle changes at the morphological level.

The presence of mucous and serous (venom) glands as the two main types, and the absence of intermediate or mixed units, suggest only two alternative developmental programmes (by epithelial-mesenchymal interactions) [101] that regulate the histogenesis of both glands. Phenotypic variability in gland structure and cell composition within individuals can arise from topographical modulation of histogenetic programmes by tissue microenvironment. Alternative developmental programmes for glands, leading to structural variability, could also be influenced by the available gene alleles, or a modulated pattern of gene expression, or to be dependent on a spatial position of glandular anlage, and influenced by local signalling or developmental noise [102] (p. 191). All these factors can lead to structural (morphological) variation, but tissue variability can also be the result of time shifts: by asynchronic (heterochronic) [103,104] developmental processes between glandular units, or between different functional stages in a secretory gland cycle. Phenotypic variability in tegumentary glands [105] over a body surface has a histogenetic basis (i.e., it is the result of local tissue interactions), but can also be secondary to local adaptive tissue changes or developmental plasticity. Hence, studying the subtle variability in gland structure has intrinsic value to evaluate the underlying flexibility of developmental programmes, to describe the functional stages in glandular units, as well as the tissue plasticity, amount of developmental, or functional noise [106].

For these reasons, the current morphological study is not about population (species’ level) phenotypic variation in tegumentary structures, but only about tissue variation within organisms, with the aim to provide an interpretation of glandular morphologies by taking into account the developmental basis of such variation, the local histophysiological dynamics, and the possible underlying mechanisms. The basic rules of developmental biology can help one to understand what could have happened to produce the phenotypical variation. It is not the common structural pattern of glands that provides hints on the causes of variation, but the subtle differences in gland architecture, cell phenotypes, and (stable or transient) cytological traits. A morphological study to identify cell types or functional stages could be performed at many levels, including histochemical markers or genetic profiling of expression, but all these must ultimately be correlated with a snapshot of the fixed (immobilised) tissue morphology that has been frozen and captured from the continuous time flow of tissue activity.

Therefore, the current histological study is about a gland’s local variation, and how it can support the hypothesis that amphibian skin can hide a topographical chemical map as variable as the colour pattern [106]. This also provides a better understanding of the use of traditional remedies in EVM. Bear in mind that this animal has been used as a therapeutic unit and therefore it will be necessary to take into account not only the “typical” composition of the perfectly developed glands but also those that are at another time in their functional cycle.

## 4. Conclusions

This histological study shows that serous/venom glands do not display a wide range of structural variability in spite of their genetic and biochemical complexity, leading to a cocktail that remains stored (and perhaps changes over time) until extrusion; in contrast with mucous glands, which work continuously to produce a surface protection layer, these also produce a set of active protein (and other) substances that dissolve into mucous material and make it a biologically active covering. As mucous glands seem to have a different size, cell composition, and kinetics in different skin regions, the mucus composition and properties can probably follow a topographical map over the skin surface. Whole skin extracts hide this specialised variation by mixing all secretions in the same pool.

From an ethnopharmacological point of view, this work provides a new view of the use of traditional remedies in EVM and human folk medicine. In fact, with this epistemological approach, with the contribution of histology, an explanation can be given to evaluate the use of toads as drugs throughout history. In this sense, our work influences from histology on how the chemical evolution of amphibian secretions can suggest new ways to fight biological agents or new toxic compounds whose mechanisms interfere in critical routes, which could be modulated by them or used as candidate drugs for the design of new drugs.

## Figures and Tables

**Table 1:** Empirical topical remedies based on the use of the natterjack toad in Spain.

	Diseases or Troubles Treated	Geographical Location	References
EVM	Scald, hoof rot	Unspecified	Charro Gorgojo [50]
	Wounds	Fuenteguinaldo (Salamanca)	González et al. [51]
	Skin ulcerations	Comarca de Zafra (Badajoz)	Penco [52]
ETHNOMED	Anthrax	Basque Country	Garmendia Larrañaga [53]
	Brucellosis	Valencia	Charro Gorgojo [50]
	Tonsillitis	Lalín (Pontevedra)	Sánchez Pérez [54]
		Galicia	Vázquez Gallego [55]
	Headache	Basque Country	Barandiaran & Manterola [56]
	Warts	Galicia	Charro Gorgojo [50], Flores Arroyuelo [57]
	Herpes zoster	Albox (Almería)	García Ramos [58]
	Venomous animal bites (spiders, snakes)	Unspecified	Flores Arroyuelo [57]

## Data Availability

All data are included as part of the manuscript.
